# T Follicular Helper Cells Mediate Expansion of Regulatory B Cells via IL-21 in Lupus-Prone MRL/lpr Mice

**DOI:** 10.1371/journal.pone.0062855

**Published:** 2013-04-24

**Authors:** Xue Yang, Ji Yang, Yiwei Chu, Jiucun Wang, Ming Guan, Xiaoxia Zhu, Yu Xue, Hejian Zou

**Affiliations:** 1 Division of Rheumatology, Huashan Hospital, Fudan University, Shanghai, China; 2 Institute of Rheumatology, Immunology and Allergy, Fudan University, Shanghai, China; 3 Department of Dermatology, Zhongshan Hospital, Fudan University, Shanghai, China; 4 Department of Immunology, Shanghai Medical College, Fudan University, Shanghai, China; 5 State Key Laboratory of Genetic Engineering, Institute of Genetics, School of Life Science, Fudan University, Shanghai, China; 6 Central Laboratory, Department of Laboratory Medicine, Huashan Hospital, Fudan University, Shanghai, China; Institut Jacques Monod, France

## Abstract

T follicular helper (Tfh) cells can mediate humoral immune responses and augment autoimmunity, whereas the role of Tfh cells on regulatory B (B10) cells in autoimmunity diseases is not clear. Here, we investigated the percentages of Tfh cells and B10 cells in lupus-prone MRL/Mp-lpr/lpr (MRL/lpr) mice and examined the effects and mechanism of Tfh cell-derived interleukin-21 (IL-21) on IL-10 production during the differentiation of B10 cells. Both Tfh cells and B10 cells were expanded in spleens of MRL/lpr mice. In addition, a positive correlation between the proportions of Tfh cells and B10 cells was observed. Tfh cell-derived IL-21 from MRL/lpr mice could promote IL-10 production during the differentiation of B10 cells. Importantly, neutralization of IL-21 inhibited IL-10 production and expansion of B10 cells both *in vitro* and *in vivo*. IL-21 induced IL-10 production via activation of phosphorylated signal transduction and activator of transcription 3 (p-STAT3). Inhibition of p-STAT3 effectively blocked IL-10 production during the differentiation of B10 cells. Moreover, IL-21-induced IL-10 exerted a regulatory function by inhibiting the proliferation of T cells. These data suggest that Tfh cells not only mediate humoral immune responses and augment autoimmunity but also play a broader role in immune regulatory actions via the induction of IL-10 production.

## Introduction

Systemic lupus erythematosus (SLE) is an autoimmune disease that involves multiple organ systems. The pathogenic mechanisms that cause lupus are unclear; however, the formation of autoantibodies against cell nuclear components is a consistent feature and is therefore likely to be fundamental to the disease [1]. The production of autoantibodies relies on T-cell-assisted B-cell activation, and thus, the immune imbalance between T- and B-cell subsets may be disturbed in SLE [2], [3], [4], [5].

T follicular helper (Tfh) cells, a CD4^+^ T-cell subset found in germinal centers (GC), express high levels of C-X-C chemokine receptor type 5 (CXCR5), programmed death-1 (PD-1), and inducible costimulatory (ICOS) molecule. These cells mainly produce IL-21 [6], [7], [8]. As the name implies, the cardinal feature of Tfh cells is their relocation to the follicular regions of secondary lymphoid tissues [9], where the cells function in the determination of whether GC B cells become memory B cells or antibody-producing plasma cells [10], [11]. Recently, circulating Tfh cells were characterized phenotypically as CD4^+^CXCR5^+^ICOS^high^PD-1^high^ cells and were expanded in peripheral blood mononuclear cells of SLE patients [12]. Sanroque mice develop lupus-like autoimmunity that is associated with greatly increased numbers of CD4^+^CXCR5^+^ T cells and enhanced expression of IL-21 [13]. In agreement with the fact that IL-21 is a key cytokine produced by Tfh cells [8], [11], we demonstrated that the genotype and allele frequencies for copy number amplifications of IL-21 were significantly higher in SLE patients than in healthy controls [14]. In the BXSB-Yaa mouse model of SLE, serum IL-21 levels increased with age and were correlated with the severity of autoimmunity [15].

IL-21 is a pleiotropic cytokine, and under certain circumstances, this cytokine exerts anti-inflammatory effects due to its ability to inhibit dendritic cell maturation and stimulate IL-10 production in T cells [15], [16]. IL-10-producing regulatory B cells (B10 cells) have recently been identified as a subset of CD19^+^CD5^+^CD1d^high^ B cells that represent 1∼3% of adult mouse spleen B cells and negatively regulate immune responses [17], [18]. The absence or loss of B10 cells exacerbates disease symptoms in contact hypersensitivity, experimental autoimmune encephalomyelitis, chronic colitis, and collagen-induced arthritis models [19], [20], [21], [22]. Tfh cells drive B cells to differentiate into plasma cells via IL-21; however, in-depth studies of the effects of IL-21 on distinct B-cell subsets, especially B10 cells, have not been performed.

MRL/lpr mice spontaneously develop a severe systemic autoimmune disease similar to human lupus, and this disease is characterized by severe pan-isotypic hypergammaglobulinemia, autoantibody production, lymphadenopathy, and immune complex-associated nephritis [23]. Although several studies showed that B10 cells were expanded in lupus-prone mice, such as MRL/lpr and NZW mice [17], [24], the reason behind this expansion of B10 cells in lupus-prone mice has not been elucidated. Here, we determined the role of Tfh cells on diverse B-cell subsets in MRL/lpr mice. We found that the percentages of both Tfh cells and B10 cells were expanded in lupus-prone MRL/lpr mice and that the expansion of B10 cells was closely related to that of Tfh cells. Tfh cell-derived IL-21 could induce IL-10 production during the differentiation of B10 cells. *In vitro* and *in vivo* neutralization of IL-21 inhibited IL-10 production and expansion of B10 cells. Furthermore, IL-21-induced IL-10 retained its regulatory function. These data suggest that Tfh cell-derived IL-21 can induce the differentiation of B10 cells and promote the production of the anti-inflammatory cytokine IL-10, which indicates that Tfh cell-derived IL-21 might be a pleiotropic cytokine. Thus, selective targeting of Tfh cells and IL-21 for the treatment of lupus requires careful consideration due to the multifactorial nature of these regulatory T cells.

## Results

### Expansion of Tfh cells in lupus-prone MRL/lpr mice

**Figure 1:**
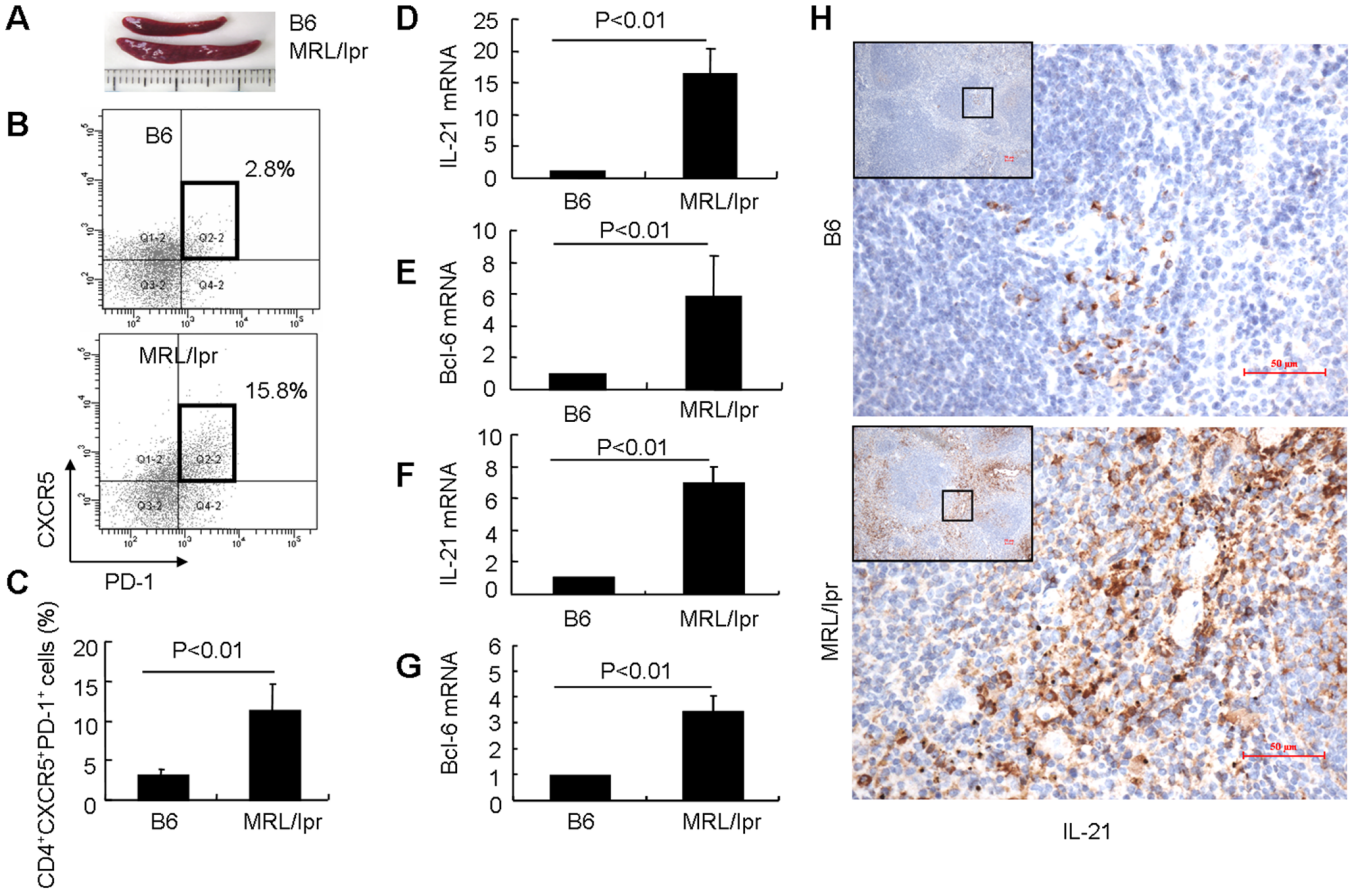
Expansion of Tfh cells in MRL/lpr mice. (**A**) Splenomegaly in MRL/lpr mice. (**B**) Splenocytes were isolated from MRL/lpr and B6 mice. After staining, cells were first gated for CD4^+^ T cells, and the CXCR5^+^PD-1^+^ cells were analyzed with a CD4^+^ gate by flow cytometry. (**C**) The percentages of CXCR5^+^PD-1^+^ cells among CD4^+^ T cells (n = 6 for each group). (**D**) IL-21 mRNA expression in fresh isolated splenocytes was determined by real-time RT-PCR (n = 6 for each group). (**E**) Bcl-6 mRNA expression in fresh isolated splenocytes was determined by real-time RT-PCR (n = 6 for each group). (**F**) Sorted CD4^+^CXCR5^+^PD-1^+^ Tfh cells from MRL/lpr and B6 mice were cultured in the presence of anti-CD3 and anti-CD28 for 2 days, IL-21 mRNA expression was determined by real-time RT-PCR. Results shown are representative of at least three independent experiments. (**G**) Sorted CD4^+^CXCR5^+^PD-1^+^ Tfh cells from MRL/lpr and B6 mice were cultured in the presence of anti-CD3 and anti-CD28 for 2 days, Bcl-6 mRNA expression was determined by real-time RT-PCR. Results shown are representative of at least three independent experiments. (**H**) IL-21 expression in spleens was confirmed by immunohistochemical staining. Further magnification of the black-bordered box shows the predominance of IL-21^+^ lymphocytes. The scale bar represents 50 μm.

MRL/lpr mice spontaneously develop a severe systemic autoimmune disease similar to human lupus [25]. At 5 months of age, MRL/lpr mice developed nephritis with increased 24-h urine protein and serious renal injuries (data not shown). Compared to age- and sex-matched B6 mice, MRL/lpr mice exhibited splenomegaly with expansion of CD4^+^CXCR5^+^PD-1^+^ Tfh cells (Figure 1A-C). IL-21 is known to be a critical cytokine produced by Tfh cells [11], and Bcl-6 is the transcription factor of Tfh cells [26]. The mRNA expression of both IL-21 and Bcl-6 was detected at high levels in splenocytes of MRL/lpr mice when compared with B6 mice (P<0.01. Figure 1D, E). Further examination revealed that IL-21 and Bcl-6 mRNA expression in sorted CD4^+^CXCR5^+^PD-1^+^ Tfh cells from MRL/lpr mice was higher than that in sorted Tfh cells from B6 mice (P<0.01. Figure 1F, G). Interestingly, the relative fold differences in Figure 1D versus 1F indicated that there was more IL-21 transcript in the MRL/lpr splenocytes than isolated Tfh cells. Other expanded T helper cells in MRL/lpr mice like Th17 cells also produced IL-21 [27], [28], which may contribute to this difference. By use of immunohistochemistry, IL-21^+^ cells were detected at higher levels in spleens from MRL/lpr mice than in those from B6 mice (Figure 1H). Examination of the expression of CD3 and IL-21 in consecutive serial sections of spleens confirmed that CD3^+^IL-21^+^ cells were present in spleens of MRL/lpr mice, but not all IL-21+ cells overlapped with CD3^+^ T cells (Figure S1). These data suggest that Tfh cells are expanded in lupus-prone MRL/lpr mice.

### Tfh cells are related to autoantibody production in MRL/lpr mice

**Figure 2:**
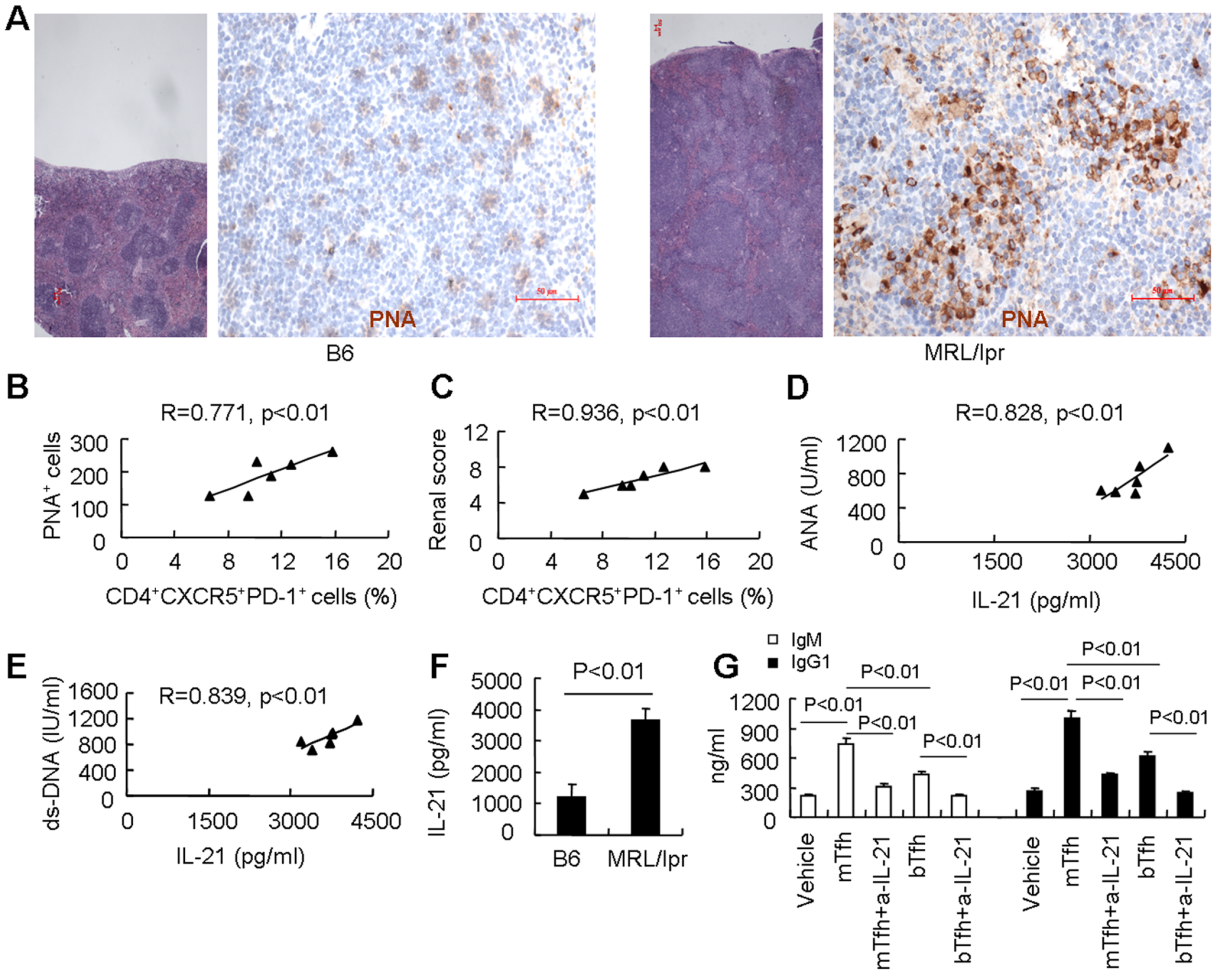
Tfh cells are associated with autoantibody production in MRL/lpr mice. (**A**) H&E staining of spleens from MRL/lpr and B6 mice (left). PNA^+^ GC cells were determined by immunohistochemical staining (right). The scale bar represents 50 μm. (**B**) A positive correlation between the percentages of CD4^+^CXCR5^+^PD-1^+^ T cells and the number of PNA^+^ GC cells in spleens of MRL/lpr mice (n = 6) was observed. (**C**) A positive correlation between the percentages of CD4^+^CXCR5^+^PD-1^+^ T cells and renal scores of MRL/lpr mice (n = 6) was observed. (**D**) A positive correlation between serum levels of IL-21 and ANA in MRL/lpr mice (n = 6) was found. (**E**) A positive correlation between serum levels of IL-21 and ds-DNA in MRL/lpr mice (n = 6) was found. (**F**) Sorted CD4^+^CXCR5^+^PD-1^+^ Tfh cells from MRL/lpr and B6 mice were cultured in the presence of anti-CD3 and anti-CD28 for 2 days, detection of IL-21 in the supernatants by ELISA. Results shown are representative of at least three independent experiments. (**G**) The concentrations of IgM and IgG_1_ in sorted naive B cells from B6 mice following a 3-day induction with LPS, anti-CD40, anti-IgM, and 20% of the supernatants from cultured Tfh cells from MRL/lpr (mTfh) and B6 mice (bTfh) or vehicle (culture media with 2 µg/ml plate-bound anti-CD3 and 2 µg/ml soluble anti-CD28) with or without neutralization of IL-21. Results shown are representative of at least three independent experiments.

Tfh cells provide selection signals that are essential for autoantibody production to GC B cells [8], [11]. Histological examination showed that peanut agglutinin (PNA)-positive GC cells were expanded in MRL/lpr mice (Figure 2A). Further analysis revealed a strong positive correlation between the percentage of Tfh cells and the number of PNA^+^ GC cells in spleens of MRL/lpr mice (R = 0.771, p<0.01. Figure 2B). In addition, the percentage of Tfh cells was also positively correlated to renal scores of MRL/lpr mice (R = 0.936, p<0.01. Figure 2C). Lupus is characterized by the overproduction of autoantibodies [1]. We found that the titers of anti-nuclear antibody (ANA) and anti-double-stranded (ds-DNA) were positively related to serum levels of IL-21 in MRL/lpr mice (Figure 2D, E). Further study showed that treatment with an IL-21-neutralizing antibody once per week for 4 weeks could inhibit the expansion of Tfh cells in spleens and reduce the titers of ANA, ds-DNA and renal scores of MRL/lpr mice (Figure S2). These data indicated that IL-21 is a promoting factor in the differentiation/expansion of Tfh cells, germinal center formation, antibody production, and autoimmunity in murine model of lupus [29], [30], [31].

As expected, Tfh cells isolated from MRL/lpr mice produced more IL-21 than those from B6 mice (P<0.01. Figure 2F), and the IL-21 intracellular expression in sorted Tfh cells from MRL/lpr mice was more than that of B6 mice (Figure S3). Interestingly, IL-21 was over-produced in old MRL/lpr mice (20 weeks of age) when compared to young MRL/lpr mice (5 weeks of age, Figure S4). Our data further showed that supernatants of cultured Tfh cells from MRL/lpr mice induced more IgM and IgG1 than that of Tfh cells from B6 mice, and neutralization of IL-21 in the culture medium abrogated the production of IgM and IgG1 (Figure 2G). These data indicate that Tfh cell-derived IL-21 may contribute to autoantibody production in lupus-prone MRL/lpr mice.

### Tfh cells are related to B10 cell expansion and IL-10 production in MRL/lpr mice

**Figure 3:**
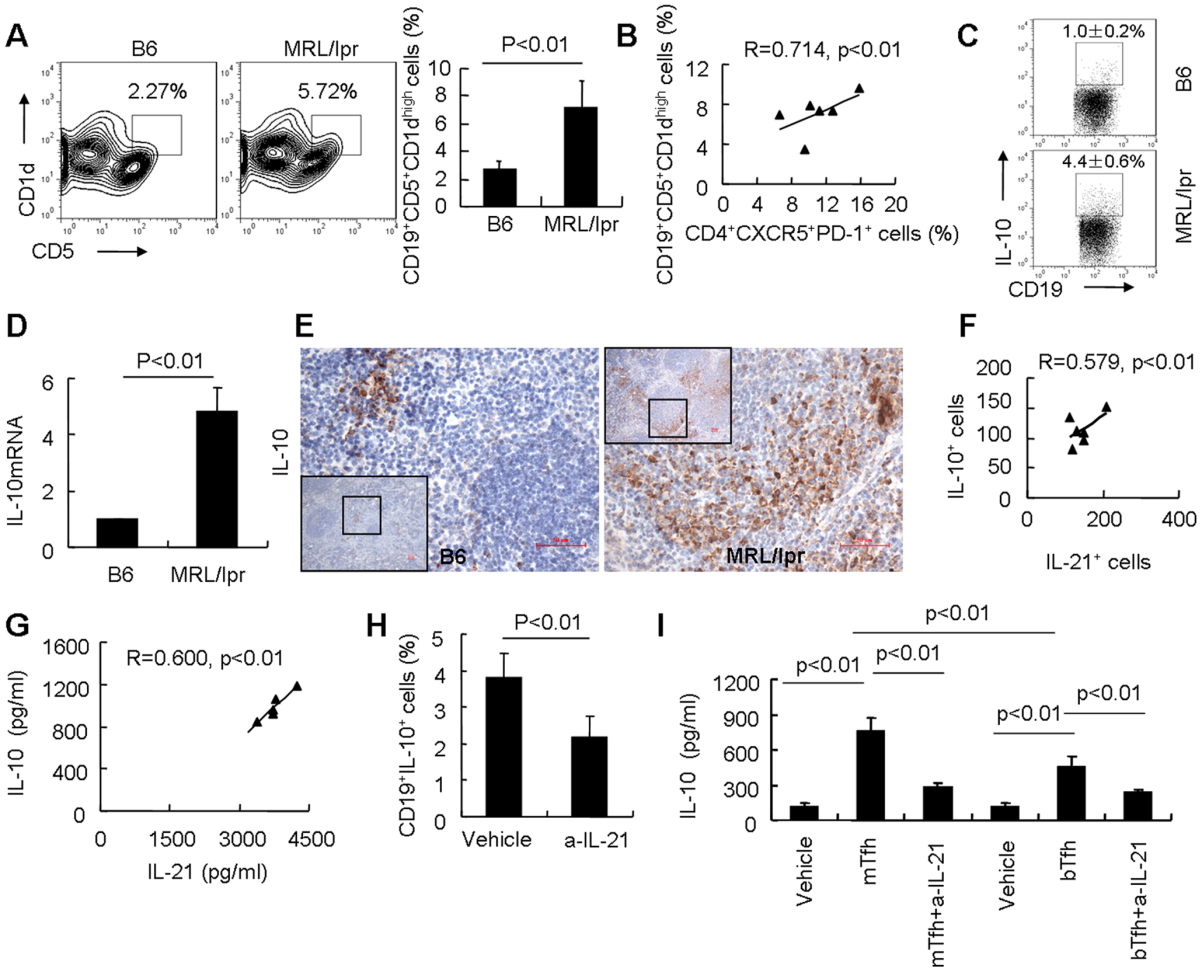
Expansion of B10 cells is positively related to increased Tfh cells in MRL/lpr mice. (**A**) Following isolation, splenocytes were stained and sorted first for CD19^+^ B cells. Then CD5^+^CD1d^high^ B cells were analyzed in a CD19^+^ gate by flow cytometry (left). The percentages of CD5^+^CD1d^high^ cells among CD19^+^ B cells are shown (right, n = 6 for each group). (**B**) A positive correlation between the proportions of CD4^+^CXCR5^+^PD-1^+^ T cells and CD19^+^CD5^+^CD1d^high^ B cells in spleens of MRL/lpr mice (n = 6) was found. (**C**) Splenocytes were isolated from MRL/lpr and B6 mice and stimulated with LPS plus PIB for 5 hours. CD19^+^IL-10^+^ cells among CD19^+^ B cells were detected by intracellular cytokine staining and flow cytometry analysis (n = 6 for each group). (**D**) Sorted CD19^+^CD5^+^CD1d^high^ B cells from MRL/lpr and B6 mice were stimulated with LPS for 48 hours and PIB for the last 5 hours, IL-10 mRNA expression was detected by real-time RT-PCR. Results shown are representative of at least three independent experiments. (**E**) IL-10 protein expression in spleens was confirmed by immunohistochemical staining. Further magnification of the black-bordered box shows the predominance of IL-10^+^ lymphocytes. The scale bar represents 50 μm. (**F**) A positive correlation between the numbers of IL-10^+^ and IL-21^+^ cells in spleens of MRL/lpr mice (n = 6) was observed. (**G**) A positive correlation between the serum levels of IL-21 and IL-10 in MRL/lpr mice (n = 6) was found. (**H**) The percentage of CD19^+^IL-10^+^ cells among B cells in spleens of MRL/lpr mice with neutralization of IL-21 or PBS vehicle control once per week for 4 weeks (n = 6) was analyzed by flow cytometry. (**I**) The concentrations of IL-10 in supernatants of cultured B6 mouse-derived B cells that were induced for 3 days by LPS plus 20% of supernatants of Tfh cell cultures from MRL/lpr (mTfh) and B6 mice (bTfh) or vehicle (culture media with 2 µg/ml plate-bound anti-CD3 and 2 µg/ml soluble anti-CD28) with or without neutralization of IL-21 and stimulated with PI for the last 5 hours were determined. Results shown are representative of at least three independent experiments.

Tfh cell-derived IL-21 promotes plasma cell differentiation and antibody production [8], whereas the effect of IL-21 on the differentiation of B10 cells, a regulatory subset of B cells with the ability to produce IL-10, is not clear. By flow cytometry, we found that the percentage of CD19^+^CD5^+^CD1d^high^ B cells was significantly increased in MRL/lpr mice (7.1±2%, n = 6, *p*<0.01) compared to B6 mice (2.7±0.6%, n = 6, Figure 3A). Furthermore, we noted a strong positive correlation between the percentage of B10 cells and Tfh cells in spleens of MRL/lpr mice (R = 0.714, P<0.01. Figure 3B). CD19^+^CD5^+^CD1d^high^ B cells were induced to express cytoplasmic IL-10 following 5 hours *in vitro* stimulation with lipopolysaccharide (LPS) plus phorbol myristate acetate (PMA), ionomycin, and brefeldin A (PIB) [17]. These CD19^+^IL-10^+^ B cells were detected at higher levels in splenocytes derived from MRL/lpr mice (4.4±0.6%, n = 6) than in those derived from B6 mice (1.0±0.2%, n = 6. Figure 3C). In addition, IL-10 mRNA expression was detected at higher levels in cultured sorted CD19^+^CD5^+^CD1d^high^ B cells from MRL/lpr mice than in cells sorted from B6 mice (p<0.01. Figure 3D). By use of immunohistochemistry, IL-10^+^ cells were detected at higher levels in spleens from MRL/lpr mice than in those from B6 mice (Figure 3E). Examination of the expression of CD19 and IL-10 in consecutive serial sections of spleens confirmed that CD19^+^IL-10^+^ B10 cells were present in spleens of MRL/lpr mice (Figure S5), but not all IL-10^+^ cells overlapped with CD19^+^ B cells. Interestingly, the infiltration of IL-10^+^ cells was positively correlated with increased IL-21^+^ cells in spleens of MRL/lpr mice (R = 0.579, p<0.01. Figure 3F). Furthermore, a strong positive correlation between the concentrations of IL-10 and IL-21 in sera from MRL/lpr mice was also observed (R = 0.600, p<0.01. Figure 3G). These data indicate that the expansion of B10 cells in MRL/lpr mice may be related to Tfh cell-derived IL-21. To test this possibility, MRL/lpr mice were treated with a neutralizing antibody to IL-21. Indeed, neutralization of IL-21 inhibited the expansion of CD19^+^IL-10^+^ cells in MRL/lpr mice (Figure 3H).

To determine whether Tfh cell-derived IL-21 from MRL/lpr mice induces IL-10 production during the differentiation of B10 cells *in vitro*, we prepared supernatants from cultured Tfh cells and examined their effects on IL-10 production. Supernatants of cultured Tfh cells from MRL/lpr mice promoted increased IL-10 production during B10 cell differentiation compared with those of Tfh cells from B6 mice, and neutralization of IL-21 in the culture medium inhibited the IL-10 secretion (Figure 3I). These data suggest that Tfh cell-derived IL-21 may contribute to IL-10 production and expansion of B10 cells in MRL/lpr mice.

### IL-21 promotes IL-10 production during the differentiation of B10 cells via activation of p-STAT3

**Figure 4:**
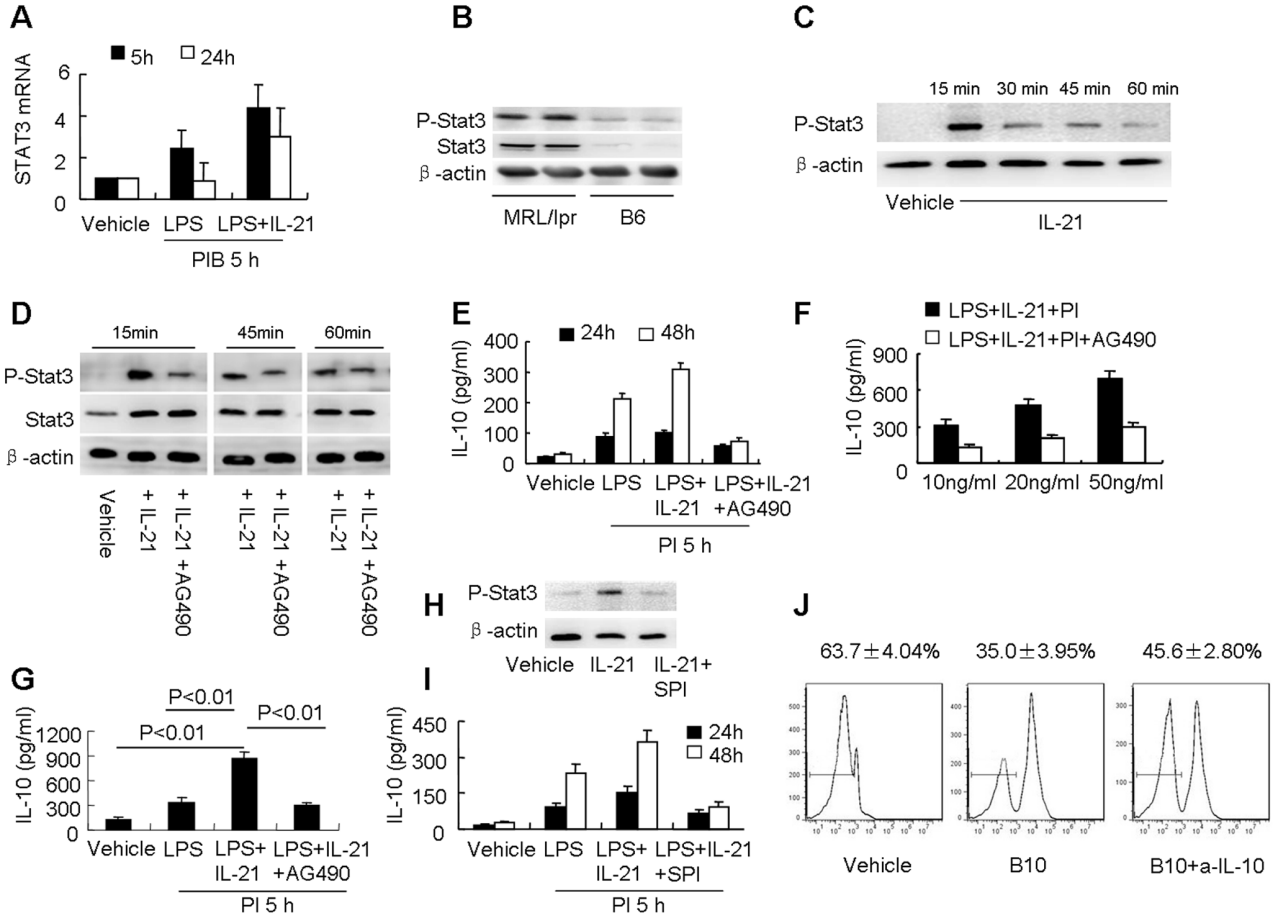
IL-21 induces IL-10 production during the differentiation of B10 cells via activation of p-STAT3. (**A**) Naïve B cells sorted from B6 mice were cultured in the presence of LPS with or without 10 ng/ml IL-21 for the indicated times and stimulated with PIB for the final 5 hours. STAT3 mRNA expression was detected by real-time RT-PCR. (**B**) The expression of p-STAT3 and STAT3 in sorted CD19^+^CD5^+^CD1d^high^ B cells from MRL/lpr mice and B6 mice was analyzed by Western blot. (**C**) Naïve B cells sorted from B6 mice were cultured with or without 10 ng/ml IL-21 for the indicated times. Then, p-STAT3 proteins were analyzed by Western blot. (**D**) Naïve B cells sorted from B6 mice were cultured with or without 10 ng/ml IL-21 or AG490 for the indicated times, and the levels of p-STAT3 and STAT3 proteins were determined by Western blot. (**E**) Naïve B cells sorted from B6 mice were cultured in the presence of LPS with or without 10 ng/ml IL-21 or AG490 for the indicated times and stimulated with PI for the final 5 hours. IL-10 in supernatants was detected by ELISA. (**F**) Naïve B cells sorted from B6 mice were cultured in the presence of LPS with or without the indicated concentrations of IL-21 or AG490 for 48 hours and stimulated with PI for the final 5 hours. IL-10 in supernatants was detected by ELISA. (**G**) Sorted CD19^+^CD5^+^CD1d^high^ B cells from MRL/lpr mice were cultured in the presence of LPS and 10 ng/ml IL-21 with or without AG490 for 48 hours, and IL-10 in supernatants was detected by ELISA. (**H**) Naïve B cells sorted from B6 mice were cultured with or without 10 ng/ml IL-21 or SPI for 15 minutes, p-STAT3 proteins were then analyzed by Western blot. (**I**) Naïve B cells sorted from B6 mice were cultured in the presence of LPS with or without 10 ng/ml IL-21 or SPI for the indicated times and stimulated with PI for the final 5 hours, IL-10 in supernatants was then detected by ELISA. (**J**) CFSE-labeled CD4^+^CD25^-^ T cells from MRL/lpr mice were cultured for 3 days with anti-CD3 and anti-CD28 antibodies in combination with 20% of the supernatants from IL-21-induced B10 cell cultures (from MRL/lpr mice) with or without neutralization of IL-10. The proliferation of these cells was determined by flow cytometry. All the above results shown are representative of at least three independent experiments.

Although our data implied that Tfh cell-derived IL-21 may contribute to IL-10 production and expansion of B10 cells in MRL/lpr mice, the mechanism of IL-21-mediated IL-10 production during the differentiation of B10 cells is not clear. IL-21 induced IL-10 mRNA expression and IL-10 secretion during the differentiation of B10 cells in a time- and dose-dependent manner (Figure S6A, B). Furthermore, IL-21 in concert with LPS promoted the differentiation of CD19^+^IL-10^+^ B cells (Figure S6C). IL-21 activates the Janus kinase/signal transducer and activator of transcription (JAK/STAT) pathway [32] and phosphorylation of STAT3 in B cells [33]; however, little is known about the role of STAT3 in the differentiation of B10 cells. We detected STAT3 mRNA expression at high levels during the differentiation of B10 cells, and addition of IL-21 augmented this STAT3 mRNA expression (Figure 4A). Expression of p-STAT3 and STAT3 proteins was detected at higher levels in sorted CD19^+^CD5^+^CD1d^high^ B cells from MRL/lpr mice than in sorted cells from B6 mice (Figure 4B), and this finding suggests that activation of STAT3 may contribute to the expansion of B10 cells in lupus-prone MRL/lpr mice.

To elucidate the role of IL-21-induced STAT3 activation in the production of IL-10, sorted naïve B cells from B6 mice were treated with IL-21 alone or combination with AG490 (a JAK inhibitor). IL-21 alone induced p-STAT3 protein expression in B cells, and AG490 effectively blocked this IL-21-induced p-STAT3 expression (Figure 4C, D, and Figure S7). This finding is consistent with previous published data indicating that AG490 inhibits p-STAT3 [34]. Furthermore, IL-21 elicited IL-10 production during the differentiation of B10 cells in a dose- and time-dependent manner, and AG490 effectively blocked IL-21-mediated IL-10 production as well (Figure 4E, F). Further study verified that inhibition of p-STAT3 by AG490 alleviated IL-21-induced IL-10 production in sorted CD19^+^CD5^+^CD1d^high^ B cells from MRL/lpr mice (Figure 4G). We further showed that SPI (a specific STAT3 activation inhibitor) could inhibit IL-21-induced p-STAT3 protein expression in sorted B cells (Figure 4H). IL-10 production could also be inhibited by SPI during the differentiation of B10 cells (Figure 4I). These data demonstrate that IL-21 may induce IL-10 production during the differentiation of B10 cells in MRL/lpr mice via activation of p-STAT3 and that inhibition of p-STAT3 blocks IL-21-mediated IL-10 production.

Although IL-10 is an anti-inflammatory cytokine [35], the regulatory functions of IL-21-induced IL-10 require examination. Proliferation of T cells in the presence of supernatants from IL-21-stimulated B10 cell culture was inhibited when compared with vehicle control, and neutralization of IL-10 restored the proliferation of T cells (Figure 4J). These data indicate that IL-21-induced IL-10 still possesses its regulatory function.

## Discussion

Our data confirmed that Tfh cells were expanded in the spleens of MRL/lpr mice. Moreover, Tfh cells were related to GC formation and renal injuries in MRL/lpr mice. IL-21, a cytokine partly produced by these Tfh cells, was overproduced in cultured Tfh cells and in sera from MRL/lpr mice, and this production was closely related to autoantibody production. Neutralization of IL-21 with an IL-21 blocking antibody could reduce autoantibody production in MRL/lpr mice. These data suggest that Tfh cells may contribute to the autoimmune pathogenesis of lupus via production of IL-21. Therefore, it is tempting to speculate that inhibitors of IL-21 may be useful to attenuate lupus-related clinical manifestations [36], [37], [38]. In designing such clinical interventions for blocking IL-21, we should, however, take into consideration not only the advantageous effects but also the risk of potential and deleterious consequences for the host. In fact, at least under certain circumstances, IL-21 also exerts anti-inflammatory effects [15], [16].

B cells can be divided into two categories: effector B cells and regulatory B cells [20], [39]. Although Tfh cells provide help for effector B cells, including memory B cells and antibody-producing plasma cells, very little is known about the role of Tfh cells in the development of B10 cells, a new subset of B cells with regulatory function. Our studies showed that B10 cells were expanded in MRL/lpr mice, which is consistent with previous research results [17]. Unexpectedly, we found a strong positive correlation between the percentages of B10 cells and Tfh cells in MRL/lpr mice. *In vitro* data also revealed that IL-21 derived from Tfh cells from MRL/lpr mice promoted IL-10 production. In addition, treatment with a neutralizing antibody to IL-21 inhibited the expansion Tfh cells and CD19^+^IL-10^+^ cells in MRL/lpr mice. Together, these data indicate that Tfh cell-derived IL-21 may be one of promoting factors for IL-10 production in lupus, which is consistent with the recently Nature published results that IL-21 is important for B10 cell development and expansion [40]. Actually, IL-21 is not only produced by Tfh cells, other T helper cells like Th17 cells also produced IL-21 [27], [28]. Further study should be done to make clear other lymphocyte-derived IL-21 on the differentiation of B10 cells. Of course, the generation of B10 subsets during autoimmune disease requires complex and reciprocal regulation; micro-environmental cytokines or other factors are also involved in the development of B10 cells [41], which needs further investigation.

The expression of p-STAT3 was detected at high levels in sorted B10 cells from MRL/lpr mice. IL-21 activates p-STAT3 [33], [42], and inhibition of p-STAT3 effectively blocked IL-21-induced IL-10 production during the differentiation of B10 cells (Figure 4E-I). These findings indicated that STAT3 may play a key role in the expansion of B10 cells in MRL/lpr mice. Previous results demonstrated that stimulation of human B cells with CD40L plus IL-21 induces IL-10 production [33], but the function of IL-21-induced IL-10 was not investigated. In our study, we presented evidence that IL-10 elicited by IL-21 provides a regulatory function by inhibiting the proliferation of T cells *in vitro*. Thus, our data expanded the prevailing belief that Tfh cells only augment autoimmunity and demonstrated that Tfh cells may function to maintain immune homeostasis by expanding the regulatory B-cell pool and stimulating IL-10 production via induction of IL-21. As a result, IL-21 antagonism is worthy of consideration for the treatment of lupus in humans. In addition, our data showed that Tfh cell culture supernatants induced comparable amounts of IL-10 compared with IL-21 used in the dose-response curve (Figure 3I, Figure S6B). We speculate that there may be other cytokines present in Tfh culture as IL-17 [43], which may act in synergy with IL-21 to affect IL-10 production during the differentiation of B10 cells. Further study should be done to make clear the complex correlation between Tfh cells and B10 cells.

Immunological homeostasis exemplifies the capacity of the immune system to upregulate immunosuppressive responses, which may ultimately limit the deterioration caused by autoimmunity. The upregulation of B10 cells may reflect a feedback regulatory mechanism that is activated to minimize harmful autoimmune responses. Our finding that IL-21-induced IL-10 production provided, at least in part, an explanation for the elevated IL-10 in lupus suggested that micro-environmental cytokines or factors may be involved in the development of B10 cells and autoimmune regulation [33], [41]. IL-10 has immunosuppressive properties related to its direct effects on various immune cells and to its ability to inhibit the production of multiple cytokines and chemokines [3]. Diminished disease severity resulted from the administration of IL-10 in the NZM2410 mouse model of lupus [44], and more severe disease occurred in MRL/lpr mice on the IL-10 KO background and in B10 cell-deficient NZB/W mice [24], [45]. Transfer of IL-10-secreting CD21^hi^CD23^hi^ B cells mitigates disease in MRL/lpr mice [46], suggesting that IL-10 derived from B cells abrogates disease in this strain. These results should also be interpreted with care, because IL-10 like IL-21 has pleiotropic effects on multiple cell lineages [45], [47], [48], [49], [50]. These diverse findings are most likely explained by the fact that the function of IL-10, either pro- or anti-inflammatory functions, depends on the applied stimuli and cell source of IL-10 [35]. In our study, we also noticed that the IL-10^+^ cells in spleens of MRL/lpr mice were not all from CD19^+^ B cells (Figure S5).

A recent study showed that B cell-derived IL-10 does not limit disease in MRL/lpr mice. In this study, however, only the IL-10 secreted by endogenous B10 cells was absent. IL-10 is typically produced by other B-cell subsets as well [47], and the progenitor B10 cells, which are a main source of IL-10 [17], [51], were not deleted in this study. Thus, insufficient data is available to solidly conclude that B10 cells do not limit disease in MRL/lpr mice [47]. In our study, we demonstrated that IL-10 induced by IL-21 still exhibited a regulatory function and inhibited the proliferation of T cells *in vitro*. These results indicated that IL-10 derived from B10 cells in response to IL-21 has the potential to recover immune homeostasis and thus offers therapeutic potential for the treatment of autoimmune diseases.

In summary, our results confirm that both Tfh cells and B10 cells are expanded in MRL/lpr mice and that Tfh cell-derived IL-21 is one of potent inducers for IL-10 production during the differentiation of B10 cells in a lupus animal model. These data indicate a new expanded role for Tfh cells and suggests that these cells can exert immune regulatory functions via promoting immunosuppressive IL-10 production. Furthermore, these data imply that lupus develops under the complex regulation of Tfh cells as well as diverse B-cell subsets, and thus, exclusive selective targeting of Tfh cells and IL-21 for the treatment of lupus may be a double-edged sword and deserves additional investigation.

## Materials and Methods

### Mice

Female MRL/lpr mice (4–5 months of age) and age- and sex-matched C57BL/6 (B6) mice were purchased from the Shanghai Laboratory Animal Center (Chinese Academy of Sciences). The onset of autoimmune diseases in MRL/lpr mice was monitored by assessment of proteinuria, and the renal score was analyzed as previously described [52]. The animal protocol was approved by the institutional animal use committee of the Shanghai Institute for Biological Sciences. All mice were maintained under pathogen-free conditions.

### ELISA

Concentrations of ANA and anti-dsDNA were determined by enzyme-linked immunosorbent assay (ELISA; R&D, Minneapolis, MN). Serum levels of IL-21 and IL-10 in mice were also detected by ELISA (eBioscience, San Diego, CA). Immunoglobulin (Ig)M and IgG_1_ in B-cell culture supernatants were detected by commercial ELISA (eBioscience). In some experiments, isolated B cells (5×10^5^ cells) were cultured and stimulated with 50 ng/ml PMA and 500 ng/ml ionomycin (PI) (Sigma-Aldrich, St. Louis, MO) for the final 5 hours. IL-10 was then detected in the supernatants by ELISA (eBioscience). Sorted CD4^+^CXCR5^+^PD-1^+^ Tfh cells (5×10^5^ cells) were stimulated with 2 µg/ml plate-bound anti-CD3 and 2 µg/ml soluble anti-CD28 (eBioscience) for 2 days, and IL-21 in supernatants was detected by ELISA (eBioscience).

### B- and T-cell isolation, culture conditions, and differentiation

Mouse naive B cells were purified by negative selection from B6 mouse spleens following the manufacturer's instructions for CD43 depletion (Invitrogen, Carlsbad, CA). For the differentiation of B10 cells, purified B cells (2×10^6^ cells/ml) were cultured in 10 µg/ml LPS (Sigma-Aldrich) for the indicated times and stimulated with 50 ng/ml PMA (Sigma-Aldrich), 500 ng/ml ionomycin (Sigma-Aldrich), and 20 μg/ml brefeldin A (PIB, eBioscience) for the final 5 hours. Experiments were performed as previously described [17], [51]. Brefeldin A was not added to cultures used for the determination of the concentrations of IL-10 in culture supernatants. Where indicated, cultures were supplemented with 10 ng/ml IL-21 for the indicated times or the indicated doses of IL-21 for 48 hours (PeproTech, RockyHill, NJ). For some experiments, B cells cultures were supplemented with the JAK inhibitor AG490 at 50 μM (Calbiochem, San Diego, CA, USA) or 50 μM STAT3 activation inhibitor SPI (BioVision, Milpitas, CA) for the indicated times. For some experiments, CD19^+^CD5^+^CD1dhigh B cells (5×10^5^ cells) were obtained via cell sorting from spleens of MRL/lpr and B6 mice and were then cultured in the presence of LPS for 48 hours and PIB for the final 5 hours for the detection of IL-10 mRNA expression. These sorted B cells were cultured with LPS+IL-21+AG490 for 48 hours and PI for the final 5 hours for the detection of IL-10 secretion. In cultures used for the detection of STAT3 and p-STAT3 protein expression, cells were not stimulated with any of these agents.

To determine the effects of Tfh cell-derived IL-21 on the activation of B10 cells, CD4^+^CXCR5^+^PD-1^+^ Tfh cells (2×10^6^ cells/ml) from MRL/lpr and B6 mice spleens were first sorted by flow cytometry, and the sorted Tfh cells were stimulated 2 µg/ml plate-bound anti-CD3 and 2 µg/ml soluble anti-CD28 (eBioscience) for 2 days. IL-21 and Bcl-6 mRNA expression in cultured Tfh cells were analyzed by real-time RT-PCR. Supernatants from these cultures were then collected for later use. Purified B cells (5×10^5^ cells) were cultured with 10 μg/ml LPS (Sigma-Aldrich) with or without 20% supernatants from the abovementioned stimulated Tfh cells (culture media with the same doses of anti-CD3 and anti-CD28 antibodies were used as vehicle control.) or 20 µg/ml anti-IL-21 neutralizing antibody (eBioscience) for 3 days and stimulated with PI for the final 5 hours. IL-10 concentrations were determined by ELISA.

To determine the effects of Tfh cell-derived IL-21 on the activation of effector B cells, sorted naive B cells from B6 mice (5×10^5^ cells) were stimulated with 10 μg/ml LPS (Sigma-Aldrich), 1 μg/ml anti-mouse CD40 (eBioscience), 1 μg/ml anti-mouse IgM (eBioscience) in the presence of 20% supernatants from the stimulated Tfh cells described above or vehicle (culture media with 2 µg/ml plate-bound anti-CD3 and 2 µg/ml soluble anti-CD28) or with 20 µg/ml anti-IL-21 neutralizing antibody (eBioscience) for 3 days. IgM and IgG_1_ in the supernatants were detected by ELISA.

To determine the effects of IL-21-induced IL-10 on the proliferation of T cells, naive B cells (5×10^5^ cells) sorted from MRL/lpr mice were first stimulated with 10 μg/ml LPS (Sigma-Aldrich) plus 10 ng/ml IL-21 (eBioscience) for 2 days and stimulated with PI for the final 5 hours. The supernatants were collected for later use. CD4^+^CD25^−^ T cells from MRL/lpr mice were first sorted by flow cytometry using fluorescein isothiocyanate (FITC)-conjugated anti-CD4 antibody and phycoerythrin (PE)-conjugated anti-CD25 antibody (eBioscience). Carboxyfluorescein succinimidyl ester (CFSE, Invitrogen)- labeled CD4^+^CD25^−^ T cells were then stimulated with 2 µg/ml plate-bound anti-CD3 and 2 µg/ml soluble anti-CD28 (eBioscience) as well as 20% supernatants from the IL-21-stimulated B10 cells described above or vehicle control (culture media with the same doses of LPS, IL-21, and PI) in the presence or absence of anti-IL-10 neutralizing antibody (10 μg/ml, R&D) for 3 days. The proliferation of T cells was analyzed by flow cytometry.

### Flow cytometry

For the detection of CD4^+^CXCR5^+^PD-1^+^ Tfh cells in mice, splenocytes were stained with PE-conjugated anti-CD4 antibody, allophycocyanin-conjugated anti-CXCR5 antibody, and FITC-conjugated anti-PD-1 antibody (eBioscience) for 15 minutes. CXCR5^+^ PD-1^+^cells were then analyzed with a CD4^+^ gate. For detection of CD19^+^CD5^+^CD1d^high^ B10 cells from mice, splenocytes were stained with PerCP/Cy5.5-conjugated anti-mouse CD19 antibody, FITC-conjugated anti-CD5 antibody, and PE-conjugated anti-CD1d antibody (eBioscience) for 15 minutes. CD5^+^CD1d^high^ cells were then analyzed with a CD19^+^ gate as previously published [17].

For intracellular IL-10 staining, splenocytes were incubated for 5 hours with 10 μg/ml LPS plus PIB. Surface staining with PerCP/Cy5.5-conjugated CD19 was first performed for 15 min and cells were re-suspended in Fixation/Permeabilization solution (Invitrogen). Intracellular staining of PE-conjugated anti-IL-10 was performed according to the manufacturer's protocol (eBioscience). After staining, CD19^+^IL-10^+^ cells were analyzed with a CD19^+^ gate. For some experiments, MRL/lpr mice were treated with an anti-IL-21-neutralizing antibody (100 µg/mouse/treatment, eBioscience) or phosphate buffer solution (PBS) vehicle control once per week for 4 weeks (from 20 weeks of age to 24 weeks of age) via intraperitoneal injection and CD19^+^IL-10^+^ cells in splenocytes were analyzed by flow cytometry. For intracellular IL-21 staining, CD4^+^CXCR5^+^PD-1^+^ Tfh cells (2×10^6^ cells/ml) from MRL/lpr and B6 mice (4 months of age) spleens were first sorted by flow cytometry, and the sorted Tfh cells were stimulated 2 µg/ml plate-bound anti-CD3 and 2 µg/ml soluble anti-CD28 for 2 days, incubated for 5 hours with PIB. Surface staining with FITC-conjugated CD4 (eBioscience) was first performed and intracellular staining of PE-conjugated IL-21 (eBioscience) was performed. After staining, CD4^+^IL-21^+^ cells were analyzed with a CD4^+^ gate.

### Western blot

B cells were cultured under the indicated conditions for the indicated times. Cells were lysed, and proteins were extracted and blotted with antibodies to STAT3, p-STAT3 (Tyr705), and β-actin (Cell Signaling Technology, Beverly, MA). The proteins were detected with SuperSignal West Pico Chemiluminescent Substrate solution (Thermo Scientific, Rockford, IL). For some experiments, the relative p-STAT3 and STAT3 protein expression were normalized to β-actin and to calculate fold-induction relative to control.

### Immunohistochemistry

Tissues were processed and stained with haematoxylin and eosin (H&E), and immunohistochemistry was performed as previously described [2]. Antibodies to IL-21 (eBiosience), IL-10, CD3, CD19 (All from Abcam, Cambridge, MA, USA), and PNA (Sigma-Aldrich) were used. IL-21^+^, IL-10^+^, and PNA^+^ cells were counted under 400 x magnification, and five independent microscopic fields were selected randomly for each sample to ensure that the data were representative and homogeneous.

### Analyses of cytokine and transcription factor mRNA expression

Total RNA was purified with the Trizol reagent (Invitrogen). Then, cDNAs were synthesized using the Primescript RT Master Mix Perfect Real-time Kit (TaKaRa, Tokyo, Japan), and mRNA expression was examined with the Bio-Rad iCycler 7500 Optical System (Bio-Rad, Richmond, CA) using a SYBR Premix EX Taq Real-time PCR Master Mix (TaKaRa). The 2^−ΔΔCt^ method was used to normalize transcription to β-actin and to calculate fold-induction relative to controls. The following primer pairs were used: Mus β-actin, forward GAGACCTTCAACACCCCAGC, reverse ATGTCACGCACGATTTCCC; Mus IL-21 forward ACAAGATGTAAAGGGGCACTGT, reverse GAATCACAGGAAGGGCATTTAG; Mus IL-10, forward CCAAGCCTTATCGGAAATGA, reverse TTTTCACAGGGGAGAAATCG; Mus Bcl-6, forward CCTGAGGGAAGGCAATATCA, reverse CGGCTGTTCAGGAACTCTTC; and Mus STAT3, forward CCGTCTGGAAAACTGGATAACT, reverse CCCTTGTAGGACACTTTCTGCT.

### Statistical analyses

Results were expressed as means ± standard deviation (SD). Statistical significance was determined by ANOVA for comparisons of multiple means, Student's *t*-test, or Mann-Whitney U-test. Correlations were determined by Spearman's ranking.
