# Correction: T Follicular Helper Cells Mediate Expansion of Regulatory B Cells via IL-21 in Lupus-Prone MRL/lpr Mice

**DOI:** 10.1371/annotation/4cdc693a-d3bb-4ca9-b094-29b061971a64

**Published:** 2013-05-14

**Authors:** Xue Yang, Ji Yang, Yiwei Chu, Jiucun Wang, Ming Guan, Xiaoxia Zhu, Yu Xue, Hejian Zou

The Supporting Information files were omitted from the article. The legends and links to files are available below.

**Figure S1. The expression of CD3 and IL-21 in consecutive serial sections of spleen.** Immunohistochemical staining of consecutive serial sections showing CD3^+^ and IL-21^+^ cells in spleens of MRL/lpr mice **(A)** and B6 mice **(B)**. Further magnification (under 400 x magnification) of the black-bordered box (under 200 x magnification) shows the typical of CD3^+^ and IL-21^+^lymphocytes.

**Figure S2. Neutralization of IL-21 reduces autoantibody production and Tfh cells in vivo.** MRL/lpr mice were treated with an anti-IL-21-neutralizing antibody (100 µg/mouse/treatment) once per week for 4 weeks via intraperitoneal injection. **(A)** The renal score of MRL/lpr mice was analyzed (n=6 for each group). **(B)** Serum level of ANA in MRL/lpr mice was analyzed by ELISA (n=6 for each group). **(C)** Serum level of ds-DNA in MRL/lpr mice was analyzed by ELISA (n=6 for each group). **(D)** The percentages of CXCR5^+^PD-1^+^ cells among CD4^+^ T cells was analyzed by flow cytometry in MRL/lpr (n=6 for each group).

**Figure S3. IL-21 intracellular expression in sorted Tfh cells.** Sorted CD4^+^CXCR5^+^PD-1^+^ Tfh cells from MRL/lpr and B6 mice were cultured in the presence of anti-CD3 and anti-CD28 for 2 days, and stimulated with PMA+ionomycin+brefeldin A (PIB) for the last 5 hours, IL-21 intracellular expression in CD4^+^ cells was detected by flow cytometry. Results shown are representative of at least three independent experiments.

**Figure S4. The production of IL-21 by Tfh cells from different weeks of age of MRL/lpr mice.** Sorted CD4^+^CXCR5^+^PD-1^+^ Tfh cells from 5 and 20 weeks of age of MRL/lpr mice were cultured in the presence of anti-CD3 and anti-CD28 for 2 days, detection of IL-21 in the supernatants by ELISA. Results shown are representative of at least three independent experiments.

**Figure S5. The expression of CD19 and IL-10 in consecutive serial sections of spleen.** Immunohistochemical staining of consecutive serial sections showing CD19^+^ and IL-10^+^ cells in spleens of MRL/lpr mice **(A)** and B6 mice **(B)**. Further magnification (under 400 x magnification) of the black-bordered box (under 200 x magnification) shows the typical of CD19^+^ and IL-10^+^lymphocytes.

**Figure S6. IL-21 promotes the differentiation of B10 cells. (A)** Naïve B cells sorted from B6 mice were cultured in the presence of LPS with or without 20 ng/ml IL-21 for the indicated times and stimulated with PIB for the final 5 hours. IL-10 mRNA expression was detected by real-time RT-PCR. Results shown are representative of at least three independent experiments. **(B)** Naïve B cells sorted from B6 mice were cultured in the presence of LPS (L) and the indicated concentrations of IL-21 for 48 hours followed by stimulation with PI for the final 5 hours. IL-10 in supernatants was detected by ELISA. Results shown are representative of at least three independent experiments. **(C)** Naïve B cells sorted from B6 mice were cultured in the presence of LPS with or without 20 ng/ml IL-21 for 24 hours. Cells were stimulated with PIB for the last 5 hours. IL-10^+^ cells were analyzed in a CD19 gate by flow cytometry. Results shown are representative of at least three independent experiments.

**Figure S7. IL-21 induces the activation of STAT3.** Naïve B cells sorted from B6 mice were cultured with or without 20 ng/ml IL-21 or AG490 for the indicated times, and the relative levels of p-STAT3 **(A)** and STAT3 **(B)** proteins were determined by Western blot. The relative p-STAT3 and STAT3 protein expression were showed as ration to β-actin and to calculate fold-induction relative to vehicle control. The data showed the quantitation of the blot bands with averages of 3 experiments. 
