# Supplementary figures and images for: Correction: T Follicular Helper Cells Mediate Expansion of Regulatory B Cells via IL-21 in Lupus-Prone MRL/lpr Mice
Source: PLoS One. 2013 May 14;8(5):10.1371/annotation/4cdc693a-d3bb-4ca9-b094-29b061971a64. doi: 10.1371/annotation/4cdc693a-d3bb-4ca9-b094-29b061971a64 (PMC3653989; doi:10.1371/annotation/4cdc693a-d3bb-4ca9-b094-29b061971a64)

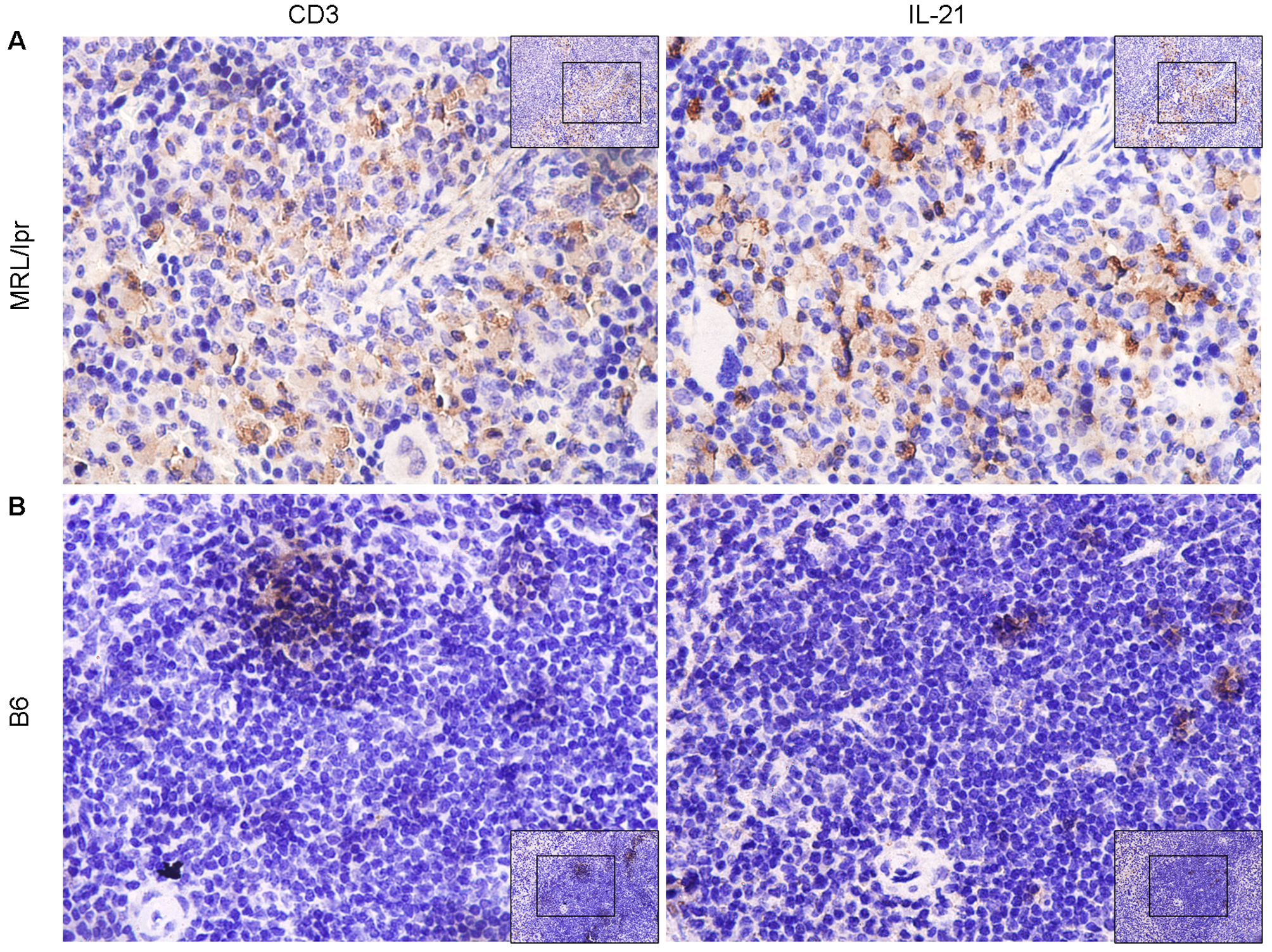

Supplement: Supplementary file 1 [file pone.4cdc693a-d3bb-4ca9-b094-29b061971a64.s001.tif]

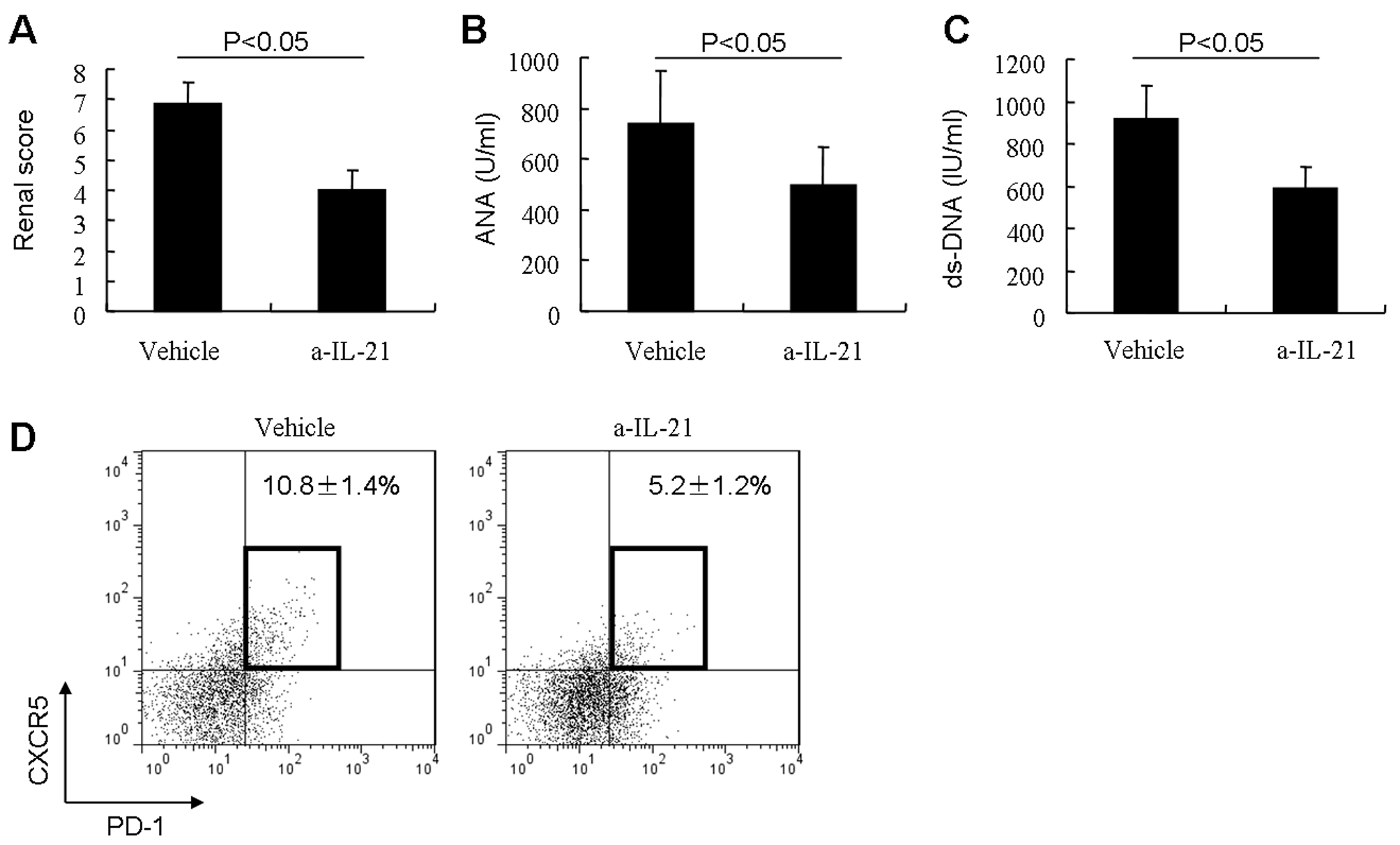

Supplement: Supplementary file 2 [file pone.4cdc693a-d3bb-4ca9-b094-29b061971a64.s002.tif]

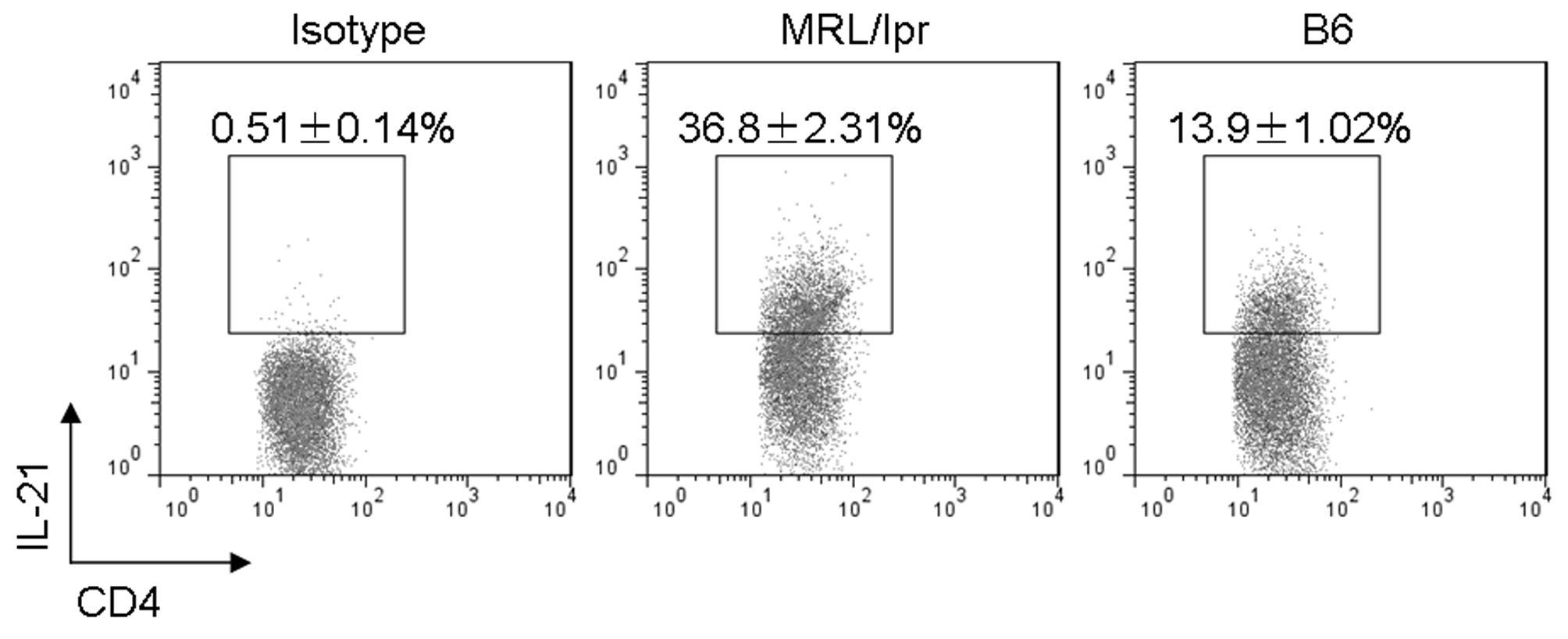

Supplement: Supplementary file 3 [file pone.4cdc693a-d3bb-4ca9-b094-29b061971a64.s003.tif]

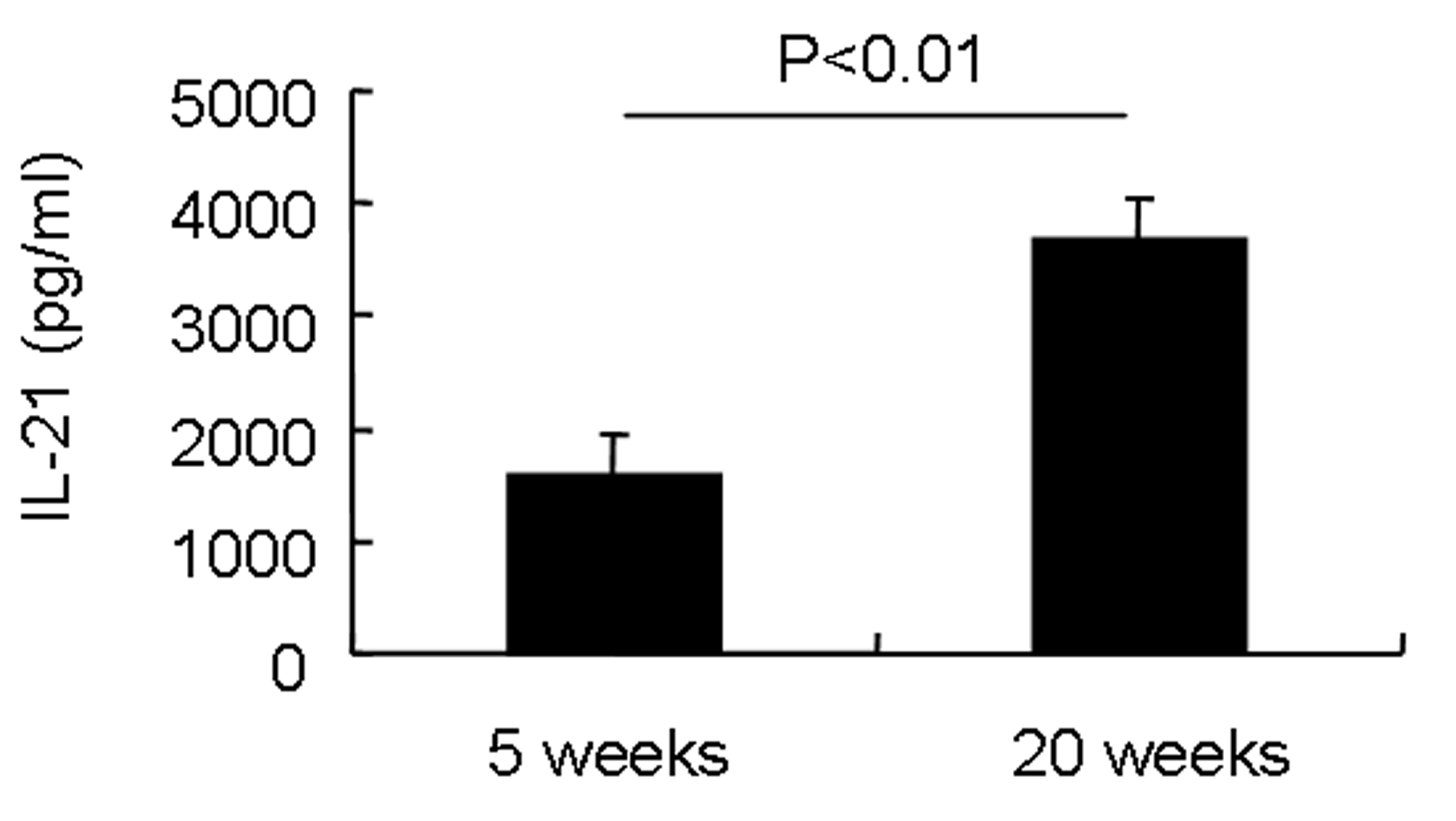

Supplement: Supplementary file 4 [file pone.4cdc693a-d3bb-4ca9-b094-29b061971a64.s004.tif]

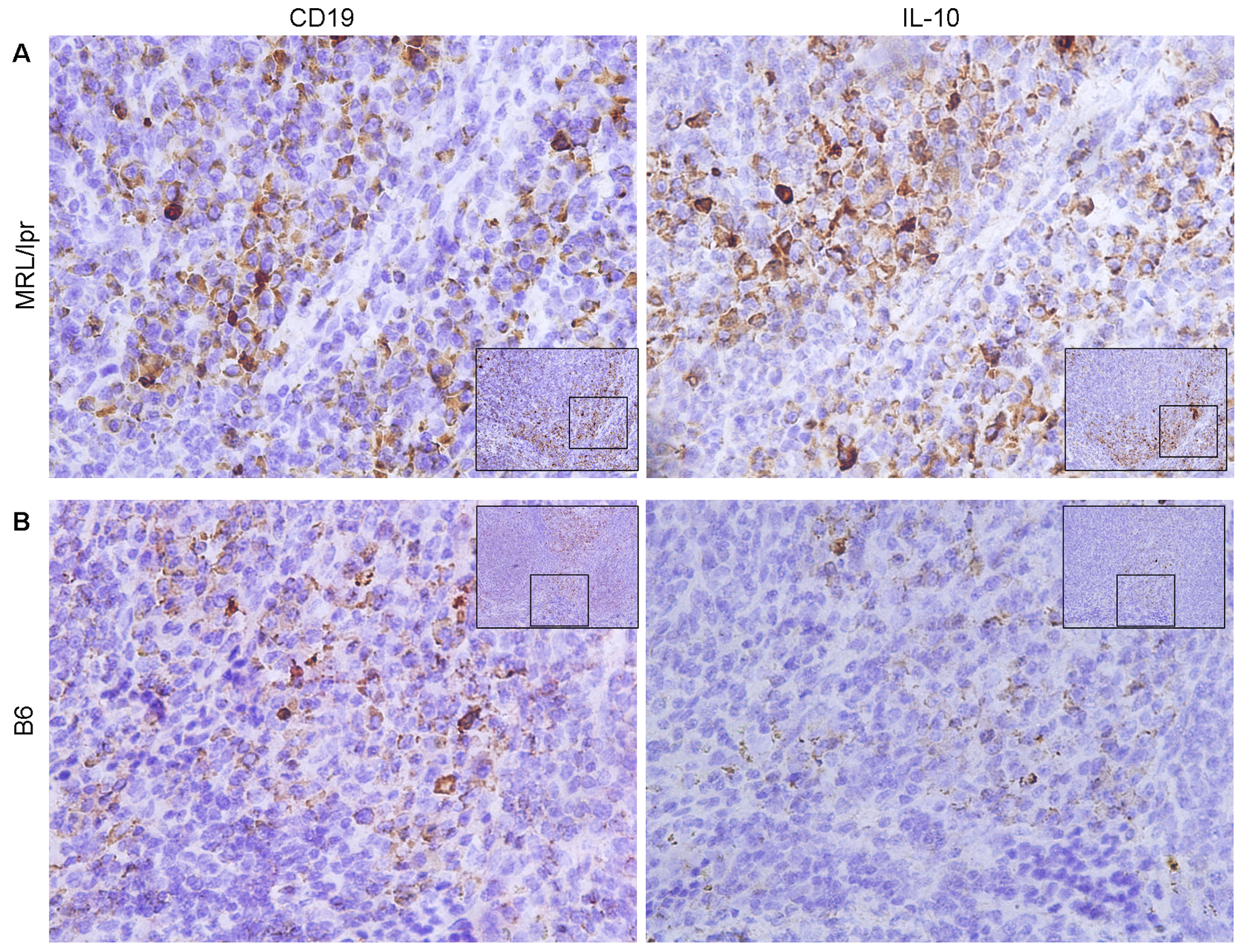

Supplement: Supplementary file 5 [file pone.4cdc693a-d3bb-4ca9-b094-29b061971a64.s005.tif]

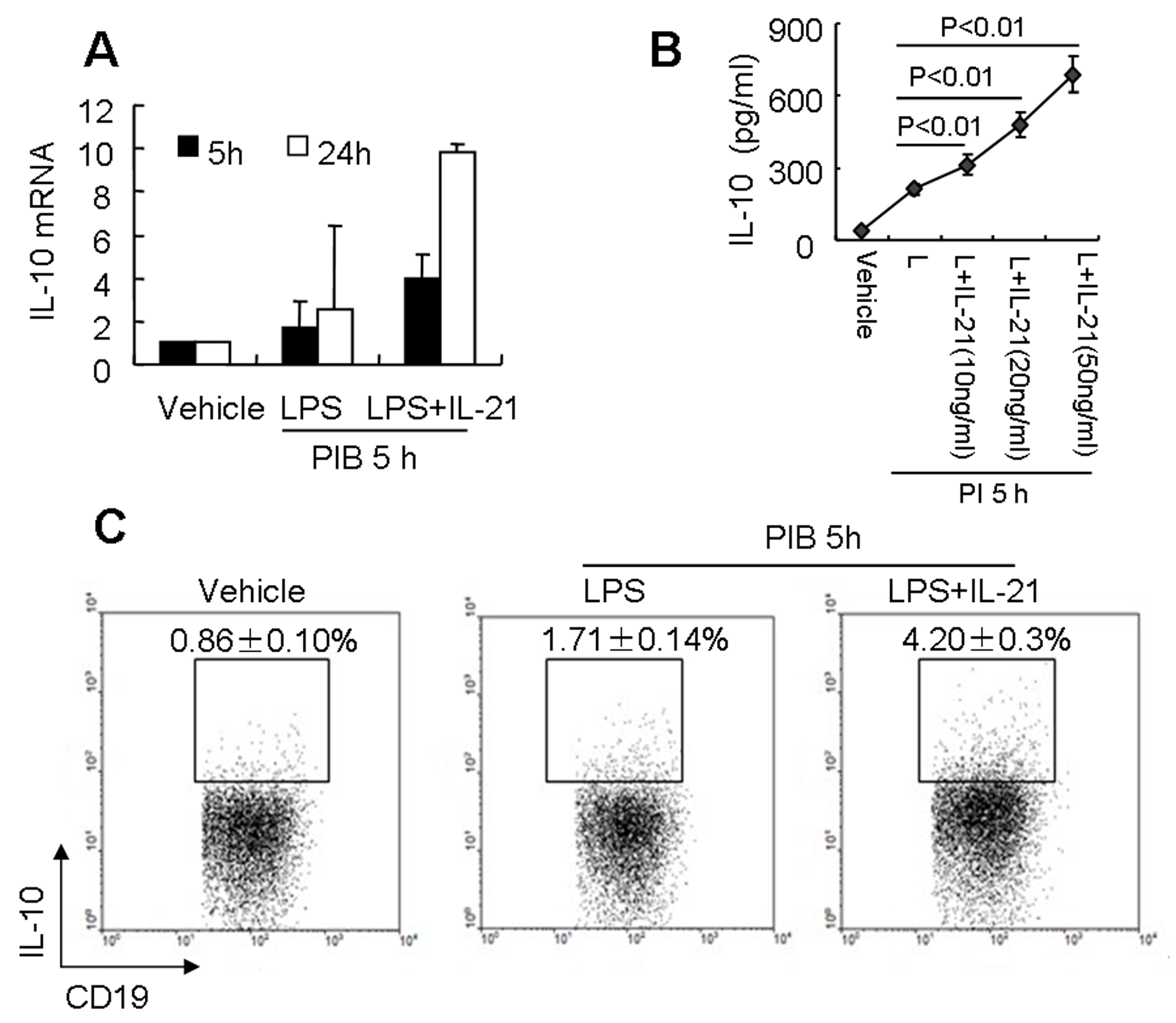

Supplement: Supplementary file 6 [file pone.4cdc693a-d3bb-4ca9-b094-29b061971a64.s006.tif]

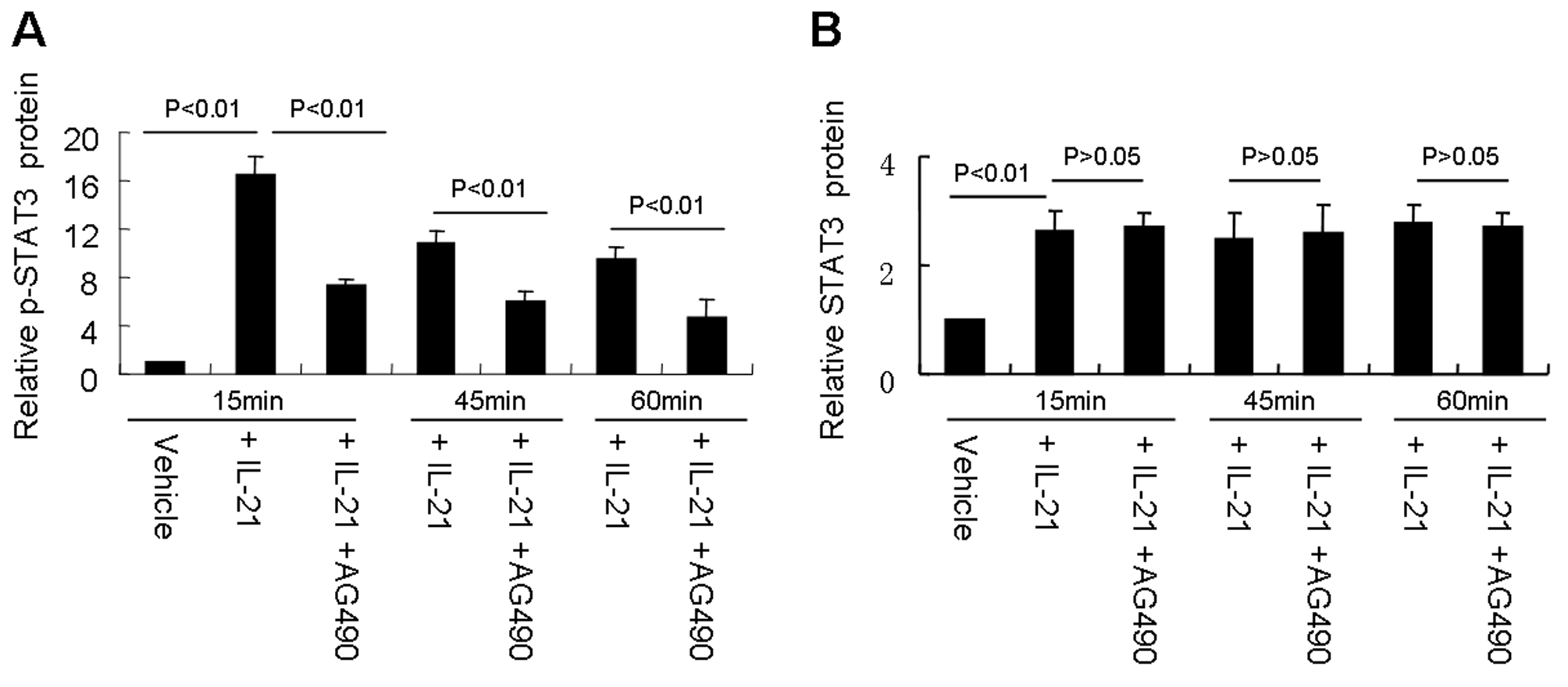

Supplement: Supplementary file 7 [file pone.4cdc693a-d3bb-4ca9-b094-29b061971a64.s007.tif]
